# Study protocol for the development, trial, and evaluation of a strategy for the implementation of qualification-oriented work organization in nursing homes

**DOI:** 10.1186/s12912-024-01883-3

**Published:** 2024-03-26

**Authors:** Corinna Burfeindt, Ingrid Darmann-Finck, Carina Stammann, Constance Stegbauer, Claudia Stolle-Wahl, Matthias Zündel, Heinz Rothgang

**Affiliations:** 1 SOCIUM Research Center On Inequality and Social Policy Mary-Somerville-Straße 3, University of Bremen, 28359 Bremen, Germany; 2 High-Profile Area of Health Sciences, University of Bremen, Bibliothekstraße 1, 28359 Bremen, Germany; 3 Department for Health Care Research, Institute of Public Health and Nursing Research (IPP), University of Bremen, Grazer Straße 4, 28359 Bremen, Germany; 4 Centre for Nursing Research and Counselling, Hochschule Bremen City University of Applied Sciences, Am Brill 2–4, 28195 Bremen, Germany; 5 Department of Evaluation and Implementation Research, aQua Institute for Applied Quality Improvement and Research in Health Care GmbH, Maschmühlenweg 8-10, 37073 Göttingen, Germany

**Keywords:** Nursing homes, Staffing ratios, Work organization in long-term care facilities, Long-term care facilities, Long-term care, Long-term care systems research, Evaluation, Organizational development

## Abstract

**Background:**

Staffing ratios in nursing homes vary among the federal states of Germany, but there are no rational grounds for these variations. In a previous study, a new instrument for the standardized calculation of staffing requirements in nursing homes was developed (*Algorithm* 1.0). The development was based on a new empirical data collection method that derives actual and target values for the time and number of care interventions provided. *Algorithm* 1.0 found an increased requirement of 36% of staff in German nursing homes. Based on these results, the German legislature has commissioned a model program to trial and evaluate a complex intervention comprising increased staffing combined with strategies for organizational development.

**Methods:**

The mixed-methods study consists of (i) developing a concept for restructuring the work organization, (ii) the application of this concept combined with increased staffing in 10 nursing homes (complex intervention), and the further development of the concept using a participatory and iterative formal evaluation process. The intervention consists of (a) quantitative measures of increased staffing based on a calculation using *Algorithm* 1.0 and (b) qualitative measures regarding organizational development. The intervention will be conducted over one year. The effects of the intervention on job satisfaction and quality of care will be evaluated in (iii) a comprehensive prospective, controlled summative evaluation. The results will be compared with ten matched nursing homes as a control group. Finally, (iv) prototypical concepts for qualification-oriented work organization, a strategy for the national rollout, and the further development of *Algorithm* 1.0 into *Algorithm 2.0* will be derived.

**Discussion:**

In Germany, there is an ongoing dynamic legislation process regarding further developing the long-term care sector. The study, which is the subject of the study protocol presented here, generates an evidence-based strategy for the staffing requirements for nursing homes.

Ethics and dissemination.

This study was approved by the Ethics Committee of the German Association of Nursing Science (Deutsche Gesellschaft für Pflegewissenschaft) on 02.08.2023 (amended on 20.09.2023). Research findings are disseminated through presentations at national and international conferences and publications in peer-reviewed scientific journals.

**Trial registration number**: German Clinical Trails Register DRKS00031773 (Date of registration 09.11.2023).

## Introduction

In most of Europe, the policy affecting the organization and provision of long-term care (LTC) has to face several socio-cultural and economic challenges [1]. Ageing societies, a shortage of skilled workers, and the aim to maintain a high quality of care are just some of them [2, 3]. There is a demand for the evidence-based development of new approaches for the organization of LTC. However, there is not only a wide range of political prerequisites but also a variety of methodological approaches measuring staffing ratios, quality of care, and, for example, job satisfaction in LTC facilities, making it difficult to evaluate the direct correlation between these parameters [4, 5].

An established way to assess the quality of care in LTC facilities is to apply quality indicators, and a growing number of countries report quality indicators publicly [6, 7]. Frequently reported indicators are related to resident safety outcomes like pressure ulcers, falls, physical restraints, and weight loss [7, 8]. In Germany, the public reporting system was revised in 2019 and now comprises the results of i) scientifically developed quality indicators [9] (Table 2), ii) external quality audits (Table 2), and iii) additional information about the nursing home (e.g., the accessibility of the care facility, the possibility of a trial stay or the staffing levels). Job satisfaction is essential to nursing home staff retention, and evidence indicates a correlation between job fluctuation and quality of care [10, 11]. Stress and low staffing levels are the most prominent reasons for job dissatisfaction among LTC staff and are still growing [12, 13].

### Objectives

This pilot program has three central aims. The first is the participatory development of a qualification-oriented work organization, trialing them combined with increased staffing (according to *Algorithm* 1.0) and evaluating the effects of this combination. The new work organization strategy is characterized by assigning work tasks individually according to the qualification of the nursing home staff (qualification orientation). The second aim is to derive a strategy for the national rollout of the implementation of qualification-oriented work organization from the evaluation findings. The third objective of the study is to refine *Algorithm* 1.0 under the condition of new work organization based on the data from the evaluation and parameterize it to yield *Algorithm 2.0*.

Our working hypothesis is that increased staffing, combined with a new work organization that matches the staff's qualifications with the resident’s care needs, can improve job satisfaction and quality of care as defined in the methods section.

## Background

This study protocol describes a study conducted by legal mandate after a Europe-wide call for tenders. The study aims to derive a strategy affecting the work organization in German nursing homes. The legal background in Germany and the underlying data collection method are crucial to understanding the study subject to this study protocol.

## Legal background in Germany

According to Paragraph 69, Sentence 1 of the German Social Code, Part 11, LTC insurance must provide needs-oriented care in Germany. That means that the provision of care must center the person’s care needs instead of, for example, centering the health care system’s resources. Determining care needs again is defined in Paragraph 14 of the German Social Code, Part 11. According to this Paragraph, the detection of care needs is based on the extent of (physical, cognitive, or psychical) independence impairments. A standardized instrument for assessing the need for LTC (‘*Pflegegutachten*’) was developed, described in Paragraph 15 of the German Social Code, Part 11. Applying this instrument, the assessors and evaluators on behalf of the LTC insurance funds (‘*Medizinischer Dienst’*) classify every person needing long-term care into one of five different care grades (with care grade one indicating the lowest and care grade 5 indicating the highest level of care dependence). Paragraph 113c of the German Social Code, Part 11 again describes the financing of staffing ratios in nursing homes. These upper limits are calculated based on the care grades so that more staff can be financed when higher care grades are present in the respecting nursing home (note that the term ‘nursing home’ in Germany refers to inpatient long-term care). In Sects. 1–3, the Paragraph distinguishes between three qualification groups for the financing of nursing home staff: i) unqualified nurses (‘*Hilfskräfte’*), ii) semi-qualified nurses (‘*Assistenzkräfte’*), and iii) qualified nurses (‘*Fachkräfte’*).

The legally defined and highly standardized procedure described in the Paragraphs mentioned above is valid in all the states (‘*Länder*’) of Germany. However, Paragraph 75 Sect. 3 of the German Social Code, Part 11 specifies that lower staffing limits must be negotiated on a state level. Due to the different financial resources of the countries, de facto staffing ratios in nursing homes vary among the federal states of Germany. However, there is no significant difference in the distribution of care grades between the states [14]. Considering that the mean time required to provide needs-oriented care derives from the care grade, staffing ratios should not vary, and there are no rational grounds for existing disparities [15].

## Qualification Levels (QL) in LTC

As seen above, German legislation applies three qualification groups to finance nursing home staff. This is based on the previous study, ‘PeBeM1’ (see below), in which a system was developed that applies the broad definitions of the National Qualification Framework (DQR) derived from the European Qualification Framework (EQF) to nursing homes [16, 17]. It creates specific definitions of Qualification Levels on a national level in the given setting (Qualification-Mix-Model; QMM). Furthermore, the QMM determines the minimum QL for the appropriate care provision for every distinct intervention included in an Intervention Catalogue [18]. The normative assignment of a targeted QL to the interventions was approved by the Quality Assurance Committee (‘*Qualitätsausschuss’*) according to Paragraph 113b of the German Social Code, Part 11.

Table 1 shows the relationship between the internationally recognized European Qualification Frame (EQF), the QL applied in the PeBeM studies, and the qualification groups of Paragraph 113c of the German Social Code, Part 11.

**Table 1:** Comparison between the European Qualification Frame, QL based on Darmann-Finck. (2021), and qualification groups of the German Social Code

European Qualification Frame (EQF)^a^	QL, according to the PeBeM study (see Darmann-Finck 2021)	Paragraph 113c of the German Social Code, Part 11
Level 1: ▪ Basic general knowledge, ▪ Basic skills required to carry out simple tasks, ▪ Work or study under direct supervision in a structured context	QL 1: Employees without training, after four months of supervised work	unqualified nurses
Level 2: ▪ Basic factual knowledge of a field of work or study, ▪ Basic cognitive and practical skills required to use relevant information in order to carry out tasks and to solve routine problems using simple rules and tools, ▪ Work or study under supervision with some autonomy	▪ QL 2 (care): 2–6-month introductory nursing course (at least 150 h)^b^ and a total of at least one year of supervised work ▪ QL 2 (supervision and support): Caregivers with 160 h lessons and three weeks internship	
Level3: ▪ Knowledge of facts, principles, processes, and general concepts in a field of work or study, ▪ A range of cognitive and practical skills required to accomplish tasks and solve problems by selecting and applying basic methods, tools, materials, and information, Take responsibility for the completion of tasks in work or study; adapt own behavior to circumstances in solving problems	QL 3: Nursing assistants with one or two years of training (according to state-specific training regulations)	semi-qualified nurses
Level 4: ▪ Factual and theoretical knowledge in broad contexts within a field of work or study, ▪ A range of cognitive and practical skills required to generate solutions to specific problems in a field of work or study, ▪ Exercise self-management within the guidelines of work or study contexts that are usually predictable but are subject to change; supervise the routine work of others, taking some responsibility for the evaluation and improvement of work or study activities	QL 4: Nursing staff with at least three years of full-time vocational training	qualified nurses
Level 5: ▪ Comprehensive, specialized, factual, and theoretical knowledge within a field of work or study and an awareness of the boundaries of that knowledge ▪ A comprehensive range of cognitive and practical skills required to develop creative solutions to abstract problems ▪ Exercise management and supervision in contexts of work or study activities where there is unpredictable change; review and develop the performance of self and others	▪ QL 5 (specialist): Nursing staff with at least 160 hours^b^ of theoretical training (e.g., palliative care, geriatric psychiatry, wound management) following the state-specific training regulations and two years of work experience ▪ QL 5 (management): Nursing specialist with further training for management tasks (at least 460 h of theoretical instruction)	
Level 6: ▪ Advanced knowledge of a field of work or study involving a critical understanding of theories and principles ▪ Advanced skills, demonstrating mastery and innovation, required to solve complex and unpredictable problems in a specialized field of work or study ▪ Manage complex technical or professional activities or projects, taking responsibility for decision-making in unpredictable work or study contexts; take responsibility for managing the professional development of individuals and groups	QL 6: Nursing specialist with a Bachelor's degree (primary qualifying degree, management studies, or similar)	

^a^Further levels 7 and 8 are part of the systems but are omitted in the comparison

^b^Note that the definition is based on Darmann-Finck 2020 but further developed

## PeBeM data collection method and *Algorithm*1.0

In 2020, a new standardized data collection method was developed and applied within the study ‘Development of a scientifically based procedure for the standardized calculation of staffing requirements in long-term care (PeBeM 1)’. The aim of the study was the development of an instrument that was able to calculate the quantity (in terms of the number) and quality (in terms of the qualification) of required nursing home staff (PeBeM is an acronym for the German word *Personalbemessung* – calculation of staffing ratios). That instrument is called *Algorithm* 1.0 [15].

The PeBeM data collection method consists of three core elements:

1. Compiling a new assessment of the need for LTC (‘Pflegegutachten’). Within the PeBeM data collection method, experts from the respective assessors and evaluators, on behalf of the LTC insurance funds (i.e., Medizinische Dienste/medicproof GmbH), apply this assessment to gain actual information about the care and health conditions of nursing home residents.
2. Preparing a care intervention plan that follows the day's structure (‘daily intervention planning’, DIP). Scientists from the study team (who also have an exam in nursing) and nurses from the respective nursing homes tailor an individual DIP for every resident. All available and relevant information about the care-dependent person is brought together. The aim of this step is the detailed depiction of the dependents' individual care needs and the planning of all necessary care interventions for the data collection period.
3. The shadowing of the nursing staff. The care provision is observed in the nursing home for about one week. While working, the facility's nursing staff is accompanied by a data-collecting shadower (also qualified nursing staff) on a one-to-one basis. The shadowers document the interventions in real-time and the time needed on an electronic device (tablet computer). The QL of the nursing staff is documented automatically by the software. All interventions planned in the DIP appear on the tablet computer screen and must be documented regarding the actual. The system gives time stamps for the duration of documentation when the shadowers start and stop the observed intervention. If an intervention is not provided, the shadowers document a reason. Additionally, they rate the time spent (in terms of surcharges or deductions) and the number of interventions (that is, they state whether a provided intervention was unnecessary or a necessary intervention was not provided).

The PeBeM data collection method is the first standardized, evidence-based data collection method that combines empirical and normative elements and provides data with information about i) number, ii) duration, and iii) qualification on both the actual and the target level. The comparison of the targeted QL (required by the complexity of the residents’ care and health status) and the actual QL from the nursing home staff that provided the care intervention derives the level of QL fitting. These levels are i) fitting (i.e., the QL is as high as required), ii) overqualified (i.e., the QL is higher than required), or iii) underqualified (i.e., the QL is lower than required). Reducing the difference between IS and OUGHT values of number, duration, and qualification can be interpreted as improving the quality of care.

### Algorithm 1.0

is a mathematical instrument that determines the required nursing home staff (in number and qualification) based on the number of residents and their care mix within a nursing home. The mean actual and targeted numbers of care interventions and time of provided care (per care dependent in a certain period) can be combined multiplicatively, and required staff numbers can be derived. The primary outcome of applying the algorithm is that for Germany, about 36% more nursing home staff are required to provide adequate needs-oriented care. The input for this calculation was the care mix of all nursing home residents in Germany. The plus of 36% full-time equivalents refers to a care-degree standardized reference nursing home with 100 residents [15]. However, the instrument addresses the overall number of required nursing home staff and specifies it according to the different QL described above. It was found that the lack of staff varies according to the QL of nursing home staff. In the legally given three qualification groups described above, the additional demand for staff in unqualified and semi-qualified nurses was 69,0%. In contrast, for qualified nurses with higher QLs, it was only 3.5% [15].

Based on the study results, the German legislature commissioned a model program to (i) trial and evaluate both measures (increased staffing and work organization), (ii) derive a concept for a national rollout, and (iii) use the findings from the evaluation to parameterize *Algorithm 2.0.* The contracting authority of this model program is the GKV-Spitzenverband (*Spitzenverband Bund der Krankenkassen* – National Association of Statutory Health Insurance Funds).

## Methods and analysis

### Study design and participants

The entire mixed-methods study is planned to run from December 2022 to May 2025 and comprises a complex intervention (running over one year) and its evaluation. The study is designed in four steps. Although in this article, the steps of the study are described consecutively, we should emphasize that in practice, they cannot be considered discrete and independent. The study steps are (i) the development of an initial implementation concept, (ii) the application of the initial concept and the co-creative, iterative development of customized concepts, (iii) a comprehensive summative evaluation, and finally, (iv) the derivation of prototypical concepts for qualification-oriented work organization which considers different starting points of nursing homes and thus offers different development paths for different types of nursing homes, the development of a strategy for the national rollout, and the parameterization of *Algorithm 2.0*.

The intervention consists of increased staffing (based on *Algorithm* 1.0) in 10 nursing homes and restructuring the work organization (intervention group). The central aim of the restructuring of the work organization is qualification orientation. That means that the workflow organization considers the qualifications of the nursing home staff and the resident's care needs to optimize the matching between these parameters (QL-fitting).

Ten matched nursing homes as a control group do not undergo the two intervention measures but participate in the evaluation. Matching criteria are the federal state, number of beds, and ownership. The evaluation assesses (a) the quality of care, (b) the job satisfaction, and (c) the effects on the actual-target differences in number, duration, and QL fit of care provision as defined in step 3 (summative evaluation). QL-fit means that the required QL of the intervention (determined by the complexity of the intervention in interaction with the level of stability of the care situation of the nursing home resident) fits the QL of the nurse. Effects of the intervention concerning (a) and (b) will be measured using a difference-in-difference approach in the intervention and control group nursing home facilities. The difference in difference analysis can be applied when evaluating outcomes associated with healthcare policy implementation. This analysis's beneficial characteristic is the possibility of controlling for background changes [19]. The evaluation compares the pre- and post-intervention differences in outcomes in (a) and (b)—as operationalized below—between the treatment group (10 nursing homes retrieving the intervention) and the control group (10 nursing homes not retrieving the intervention). Endpoint (c) will only be investigated through a pre-post comparison with the intervention group, as the shadowing of the control group was regarded as too resource-intensive.

The study will involve 20 nursing homes in Germany (10 in the intervention group and 10 in the control group), nursing home staff, residents, about 120–150 data collectors (so-called ‘nurse shadowers’), and about 20 assessors and evaluators on behalf of the LTC insurance funds (Medizinische Dienste/medicproof GmbH). The respective work packages describe the participants' involvement in detail.

## Eligibility criteria

Several targeted groups are involved in the study. Nursing home staff must fulfill partially varying inclusion criteria for participation in the different activities. For the survey, they must be involved in the care of the respective nursing home residents and working in the home before the start of the survey. For the shadowing, they must work in the shadowed living area (only in the intervention group). The focus groups must be employed in a participating nursing home from the beginning of the intervention (only in the intervention group). For the competency analysis, they must work in one of the nursing facilities of the intervention group.

Nursing home residents may participate in the survey if they can complete the questionnaire alone or with assistance. A proxy rating will be applied for nursing home residents with cognitive impairments. Participation in the shadowing is possible for nursing home residents if they live in the respective shadowed living area. Terminal residents and residents living in the respective nursing home for less than four weeks will be excluded from the shadowing and the survey.

The inclusion criteria for the shadowers participating in data acquisition (i.e., shadowing) are registration as a qualified (geriatric) nurse and participation in training provided by the study team. There are no further inclusion criteria for the shadowers' survey.

## Recruitment

All nursing homes in Germany that submitted a written statement of interest in participating in the study before a deadline could apply. The study team preselected 30 facilities (concerning the state, number of beds, and ownership). These nursing homes have an average capacity of 76 beds (between 30 and 120 beds), are distributed in all 16 states, and between non-profit, public, and private organizations according to the distribution in Germany. Rural and urban areas were taken into account. After visiting the premises and talking to the staff, the research team forwarded the names of 20 nursing homes to the GKV-SV as the study's funder, which made the final selection of the ten nursing homes for the intervention group. The GKV-SV then concluded a subsidy agreement with the selected nursing home facilities. The nursing homes of the control group are selected by the study team and reviewed by the GKV-SV.

Participation in the study is voluntary and based on informed consent. Participants of the intervention group will be recruited at information events held by the study team in the participating nursing homes. At these information events, the study team aims to inform the staff, the residents, and relatives about the study's aims and procedure, enlist support, and recruit survey participants. Participants for the control group are informed and recruited by the respective nursing home management.

**Fig. 1:**
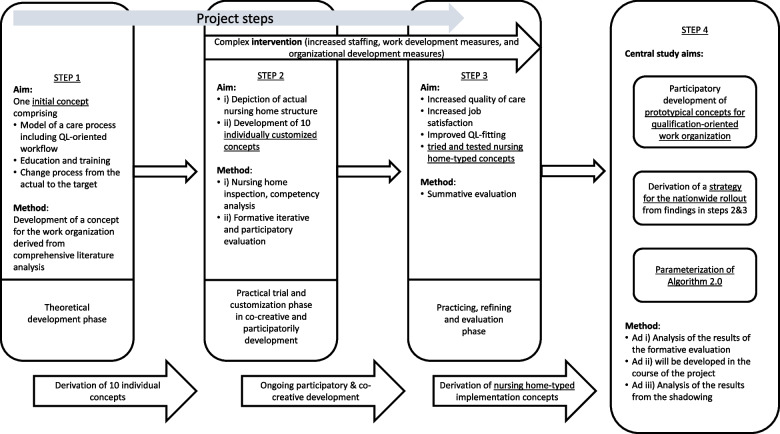
Conceptual framework of the mixed-methods study

The study is structured into four study steps (Fig. 1).

## Development of an initial concept

In the theoretical development phase, an initial concept for implementing a qualification-oriented work organization in nursing homes will be developed, starting with a comprehensive national and international literature analysis. Literature will be considered concerning nursing processes, education and training in nursing homes, and theoretical conceptualizations of change processes in organizations. The theoretical basis for the management measures derives from diverse sources in organizational development, personnel development, and change management theories. The management measures will include, for example, monitoring the QL fitting and developing change-process plans. The following components will be combined into the initial concept:

i) A nursing process that centers on the care needs of nursing home residents.
ii) Education and training measures for different QLs. Formal training courses are planned for QL 4. Training courses are being developed at a low threshold in the QL 1 and 2 work process.
iii) A theoretical model for the organizational change process aims for a qualification-oriented work organization. That means a strategy will be developed to assess the QL in nursing homes (actual and target) and adjust the workflow to increase QL fitting.

## Application and co-creative, iterative development of customized concepts

As one single concept cannot be appropriate for all different nursing homes, in the practical trial and customization phase, the initial concept will be further developed into customized and empirically refined implementation concepts. The intervention will begin with applying the initial concept in the ten facilities of the intervention group. The intervention comprises a quantitative and a qualitative component: firstly, increased nursing home staffing based on the previously developed *Algorithm* 1.0 [15]. Secondly, qualitative measures which include organizational (work organization with the aim of QL-fitting) and personnel development (education and training). The iterative development process from the initial to customized concepts starts as the intervention begins. The method applied for further iterative development is formative evaluation. This will be conceptualized as an ongoing participatory process of co-creation by scientists and practitioners from nursing homes.

It starts with depicting the actual status to set an individual starting point for the change process. The assessment of the actual status of the nursing homes comprises:

i) Nursing home inspections regarding organizational structures and management of the homes (e.g., infrastructure, care and case mix, organizational concepts), person-related conditions (e.g., staff and nursing home residents), and service provision processes.
ii) An assessment for the analysis of competencies on the individual level of the nursing homes’ staff. The analysis of competencies is composed of (i) a self-assessment of competencies for nursing home staff, (ii) a proxy assessment of competencies for management staff, and (iii) a comparison of the results in a personal development meeting. Educational needs can be derived from the comparison results and the agreements made during the talks. The instruments will be developed during the project [20]. The reliability of the self- and proxy assessments will be tested by calculating Cronbach’s alpha.

The formative evaluation process will comprise three instruments to evaluate the concept components' effectiveness, acceptance, and practicability. The instruments are:

i) Research diary: Research team members with direct contact with the facilities regularly fill out a standardized form for each nursing home (timely after contact, at least every two weeks). The form contains questions about events, experiences, and information that result from the contact and can provide recommendations for the further development of the concept.
ii) Semi-structured expert interviews: Every two weeks, an interview will be conducted with a contact person at the facility (usually the home or nursing service management). Nursing home-specific findings from the research diaries will be used to customize the interview guides and to find solutions co-creatively. In addition to these individual topics, the interviews will concern, e.g., challenges associated with the implementation of the work organization, perceived changes, and the identification of resource-intensive work steps in the implementation.
iii) Focus groups: Every two weeks, members of the study team, with direct contact with the facilities, combine the results of the research diary entries and the expert interviews to develop recommendations for further developing customized concepts.

The three elements are repeated continuously for one year, creating an iterative further development and customization process.

## Summative evaluation

The customized concepts will be practiced, refined, and evaluated in the third study phase. The evaluation is planned as a prospective interventional study. In the intervention group, all elements of the data acquisition for the summative evaluation will take place as pre-post-comparison at two points in time (t_0_ before the intervention and t_1_ after the implementation of the changes in organization and staffing) at an interval of one year (note that the shadowing only takes place in one living area per care home). To control for general trends in the dynamic LTC setting and the effects of the revision of Paragraph 113 Section C of the German Social Code, Part 11 (that enacted a new finance of staffing ratios based on *Algorithm* 1.0) 10 nursing homes from the intervention group are matched with ten nursing homes in the control group by state, number of beds, and ownership. The control group undergoes the surveys of the summative evaluation but not the shadowing nor the two intervention measures. A (cluster) randomization was not possible due to the stipulations of the statutory order.

The summative evaluation assesses the effects of the intervention on (i) quality of care, (ii) job satisfaction, and (iii) changes in the difference between (a) the number of provided interventions, (b) the duration of the provided interventions, and (c) the extent of qualification-fitting. Table 2 provides an overview of the data collection methods for the endpoints of the summative evaluation.

**Table 2:** Data collection methods for the endpoints of the summative evaluation

Methods and instruments	Operationalization of the endpoints
	**Quality of care**
1. Secondary data analysis of: a) Obligatory and publicly reported quality indicators, b) Results of external quality audits	a) Self-assessed 15 indicators regarding the three themes: 1. Maintaining and promoting independence (e.g., in mobility), 2. Protection against health hazards and stress (e.g., in the development of pressure ulcers, falls, unintended weight reduction), 3. Support for specific needs (e.g., timeliness of the pain assessment, interaction with cognitively impaired residents) are assessed (percentage of residents with b) External quality audits assess 15 items regarding the four themes: 1. Support with mobility and self-care (e.g., support with eating and drinking, mobility, continence loss, continence promotion, personal hygiene, taking medication), 2. Support in coping with the demands and stresses of illness and therapy (e.g., taking medication, pain management, wound care), 3. Support in organizing everyday life and social contacts (e.g., impaired sensory perception, structuring the day, occupation and communication, nocturnal care), 4. Support in special needs and care situations (e.g., Support during the settling-in phase after moving in, transition management during hospitalization, support for residents with challenging behavior, use of measures involving deprivation of liberty)
2. Standardized survey of nursing home residents: a) ASCOT questionnaire b) EQ-5D and c) Proxy rating	a) Subjective care-related quality of life of nursing home residents is assessed in 8 dimensions: 1. Self-determination, 2. Personal hygiene and clothing, 3. Food and drink, 4. Feeling safe, 5. Talking and being together, 6. Spending time doing things you enjoy doing, 7. Clean, cozy home, 8. Influence of caregivers on self-esteem b) 5 dimensions of the health-related quality of life: 1. Mobility, 2. Self-care abilities, 3. Everyday activities, 4. Pain/ physical complaints, 5. Anxiety/depression c) 40 items in 10 subscales: 1. Relationship to nursing home staff^c^, 2. Positive affect^c^, 3. Negative affect^c^, 4. Restless/ tense behavior, 5. Positive self-image^d^, 6. Social relationships^c^, 7. Social isolation, 8 Feeling at home^d^, 9. Having something to do^d^, 10. Items without subscale
3. Standardized evaluation of shadowing data	PeBeM-indicators: 1. Actual number of provided interventions, 2. Targeted number of provided interventions, 3. Actual duration of the provided interventions 4. Targeted duration of the provided interventions 5. Actual extent of qualification fitting 6. Targeted extent of qualification fitting
4. Partially standardized survey of the shadowers	Qualitative assessment of the perception of care provision. The assessments will be developed and contain questions regarding: 1. Perception of communication and collaboration, 2. Perception of the interaction between nursing home staff and residents, 3. Perception of quality of care 4. Perception of the work organization
	**Job satisfaction**
Standardized survey of employees: a) Employee Experience Questionnaire (EXQ), b) StressBarometers	a) Two subscales with overall nine items regarding: 1. General job satisfaction (i.e., development opportunities, colleagues, work, management, remuneration), 2. Organizational commitment (i.e., concern for the institution's future, praise for the institution, pride in belonging to the institution, goodness of the employer) b) Three subscales regarding: 1. Organization and time management (4 items), 2. Tasks and content (20 items), 3. Collegial communication and exchange within the work (10 items)
	**Changes in actual-target difference**^**b**^
Shadowing^a^	Number of provided interventions Duration of the provided interventions Extent of qualification fitting

^a^ conducted in one living area in each facility from the intervention group

^b^ See Table 3

^c^Some of the items in the subscale are only applicable in QUALIDEM I (and not in QUALIDEM II for severe cognitive decline) ^d^All of the items of the subscale are only applicable in QUALIDEM I (and not in QUALIDEM II for severe cognitive decline)

Four different approaches are applied to assess the quality of care. A secondary data analysis of obligatory quality indicators will be conducted as a first step. In Germany, since 2022, these indicators have been reported publicly in every nursing home. As shown in Table 2, two different instruments were used: firstly, there will be an analysis of the 15 publicly reported self-assessed quality indicators. Secondly, the publicly reported results from the qualification audits from assessors and evaluators on behalf of the LTC insurance funds (i.e., Medizinische Dienste/medicproof GmbH) will be analyzed. These external quality audits collect data on 15 items in 4 themes (Table 2).

Secondly, paper-based surveys of nursing home residents will be carried out based on the ASCOT questionnaire (Adult Social Care Outcomes Toolkit) on their subjective quality of life [21, 22] and the EQ-5D about health-related quality of life in an evaluated German version [23]. The ASCOT is a care-related quality-of-life instrument with a high construct validity in the given setting [24]. To be able to record changes perceived by nursing home residents due to project-specific interventions (e.g., personnel and organizational changes in the care facility) that are not recorded in the ASCOT and the EQ-5D questionnaires, questions specially formulated for the project are added to the questionnaire (e.g., on satisfaction with organization and communication, perception of turnover of nursing home staff, etc.). In the t_1_ survey, further questions are included to identify the effect of the change, e.g., ‘Has anything changed as a result of the restructuring in the last year?’. For cognitively impaired nursing home residents, a proxy rating is provided via the QUALIDEM questionnaire [25, 26]. In German, the QUALIDEM is available in two versions according to the severity of cognitive decline (measured by the nursing home staff using the Global Deterioration Scale [27]). While QUALIDEM I can be applied in nursing home residents with mild cognitive decline (GDS Stadium 2–6), QUALIDEM II is used in residents with severe cognitive decline (GDS Stadium 7). The subscales of the QUALIDEM are shown in Table 2. The written survey and the proxy rating are planned for all nursing home residents living for at least four weeks in one of the participating nursing homes in both the intervention and the control group. The eligibility criteria are stated above. The surveys will be carried out in all living areas of the respective nursing homes.

Thirdly, empirical data will be collected in one living area of each nursing home of the intervention group in an observational study (‘shadowing’). The methodology was developed in the first PeBeM study and described in the final report [15]. The three elements of the data collection method are described in the background section. To assess actual-target differences (and their changes from t_0_ to t_1_), the shadowing data will be analyzed about (i) the number of interventions provided, (ii) the duration of the interventions provided, and (iii) the extent of qualification fitting. The determination of the respecting values is shown in Table 3.

**Table 3:** Determination of actual and target data of care provision in the shadowing

Information provided in the data	Determination of the actual value	Determination of the target value	Involved Determiner
**Number** of provided interventions	Electronic time stamp while shadowing	1. DIP	1. Members of the study team qualified as a nurse
		2. Shadowers assessment	2. Shadowers
**Duration** of the provided interventions	Electronic time stamps while shadowing	Shadowers assessment	Shadowers
Extent of **qualification fitting**	Automatic electronic documentation while shadowing (data link in software between nursing staff and QL)	1. Software derives automatically from BI	1. Assessors and evaluators on behalf of the LTC insurance funds
		2. Software derives automatically from DIP (if additional information was registered)	2. As nurse-qualified members of the study team determine QL requirements individually

Fourthly, a partly standardized online questionnaire will be developed to assess the shadower's perception of the cooperation and communication structures within the nursing homes. The survey will be created based on experience from the PeBeM1 project.

Online surveys of the nursing staff will be conducted to assess job satisfaction. The standardized instruments used are the EXQ (Employee Experience Questionnaire) and the StressBarometer [28, 29]. The EXQ consists of four dimensions that address different aspects of job satisfaction. The two dimensions chosen for the study are i) overall job satisfaction and ii) organizational commitment. The other two dimensions addressing individual and collective engagement were excluded to minimize the time spent filling in the questionnaire and to increase the chances for participation in the survey. The EXQ was chosen because it is a standardized and evaluated instrument of good psychometrical quality (Cronbach’s alpha = 0.79—0.91, McDonald’s omega = 0.77 – 0.91), and it is easy to understand even if one is not a native speaker [28]. Furthermore, one of the dimensions directly addresses organizational commitment. As the intervention group in this study changes the work organization, these parameters are highly interesting for evaluating the intervention.

The StressBarometer was developed by the Federal Institute for Occupational Safety and Health (Bundesanstalt für Arbeitsschutz und Arbeitsmedizin – BauA) to record and assess psychological workloads within a job and was evaluated for many branches [29]. This instrument was chosen because it is easily understandable for non-native speakers. The utilization of the instrument in this study and the customization of the questionnaire (three subscales were selected) was permitted by the holder of rights (trade union ‘IG Metall’). The satisfaction in correlation to the restructuring of the work organization will be evaluated retrospectively at the facilities of the intervention group. The questions will be formulated during the project, taking into account the measures implemented for personnel and organizational development along with the implementation and service outcomes of the Proctor framework [30].

The focus groups, of which four are planned, are conducted after the other evaluation results have been analyzed. The focus groups aim to investigate (i) beneficial and inhibiting factors for the implementation process, (ii) positive and negative experiences during the process, and (iii) whether any practicable problem-solving scenarios can be taken into account when refining the algorithm. The guidelines for the focus groups will be based on the evaluation results and can, therefore, only be developed after the t_1_ survey.

## Prototypical concepts, strategy for a national rollout, and Algorithm 2.0

The study has three superordinated aims. The first is the participatory development of prototypical concepts for qualification-oriented work organization. According to individual nursing home characteristics like the care organization model, competency profiles of the staff, or the extent of digitalization, typed concept variants will be derived. The prototypical concepts thus consider different starting points of nursing homes and offer different development paths for various nursing homes. The definitive attributes that distinguish nursing homes with comparable preconditions will be analyzed using the iterative formal evaluation. These concepts, after rollout, can serve as templates for any nursing home from those to choose the most appropriate. Once one of the typed concepts is selected, any nursing home is free for individual adaptation. Due to the small sample size, the methodology for the analysis must be qualitative. However, the structure of the formative evaluation will take shape during the project. The final nursing home-typed concepts include procedures for a standardized census of the actual status as well as instruments and materials that depend on the prototype of the nursing home. The second final objective of the study aims to incorporate all the results and findings from project steps 1–4 and the contemporaneous participatory co-creative processes to parameterize *Algorithm 2.0*. Data from the shadowing (particularly the number of staff and data on the targeted duration of specific tasks and procedures) are analyzed to refine the algorithm. New aspects compared to *Algorithm* 1.0 will be (i) the incorporation of QLs 5 + and other professions in the calculation and (ii) target time values for indirect care and occasional care interventions. The third final objective is to develop a strategy for the national rollout. Methodological and substantive aspects for developing the nationwide rollout must be created during the project. The method under consideration is focus groups.

## Statistical analysis

No data will be collected during the development of an initial implementation concept, and no statistical analysis will be conducted. In the second work package, the actual status of the nursing homes, e.g., the infrastructure, the resident structure, the organization of the nursing homes, technical equipment, and the competencies of the staff, will be depicted. Information regarding the actual status will be analyzed descriptively.

The summative evaluation first analyzes the results of the mandatory external quality assessments descriptively. The analysis examines the development of the quality of care over the intervention period (t_0_, t_1_) in a pre-post comparison, both for the pertinent nursing homes and the control group, and the differences in the trends between them (difference-in-difference approach). Secondly, a descriptive analysis of the surveys on nursing home residents will be carried out. The analysis of the job satisfaction survey is carried out for the two subscales of the EXQ according to the method specified in the instrument. The subscales of the StressBarometer are not analyzed according to the specified scoring for workload but only in a descriptive comparison of the aggregated subscales and the individual items between the intervention and the control group and, in particular, for the indirect measurement of change in the intervention group (pre-post comparison). The analysis will examine changes in job satisfaction in t_1_ compared to t_0_ and if these changes differ between the intervention and control groups. Stratified evaluations are planned according to occupational groups and QLs. The answers to the questions on direct change measurement (t_1_ survey) will be analyzed quantitatively as far as possible; qualitative data will be summarized narratively if necessary. From the shadowing data of the intervention group, rates of the number and duration of the (non-)performance of the planned and the performed interventions are calculated (actual and targeted quantitative aspects of care). Furthermore, the change between t_0_ and t_1_ will be shown. Moreover, the analysis will examine the extent of qualification-oriented work organization and the change between t_0_ and t_1_ (actual and targeted qualitative aspects of care). The survey of the shadowers will be evaluated descriptively concerning the quality of care, possible causes of any deficits observed, and perceived changes due to the intervention.

The formative evaluation will be analyzed qualitatively due to the small sample size.

## Discussion

In Germany, in July 2023, a revision of Paragraph 113 Section C of the German Social Code, Part 11, was enacted to reform the refinancing of staffing in LTC. The calculation of staffing ratios applied in this paragraph is based on *Algorithm* 1.0, which resulted from the PeBeM1 study. During the ongoing dynamic process of legislation regarding the further development of the LTC sector, the PeBeM3 study now investigates a combination of staffing calculation and work organization to generate an evidence-based strategy for elaborating the care system. Socio-cultural and economic challenges in the organization and provision of LTC affect most of Europe. Thus, developing new work organization and resource distribution strategies is relevant for further developing healthcare systems. Furthermore, a qualification-oriented work organization has yet to be developed and evaluated in the given setting. It could be transferred to and implemented in other healthcare systems. Therefore, the study's results will be considerable for nursing sciences – not just in Germany. For experts in different countries, the study will hopefully serve as a template to restructure LTC financing or work organization.

## Limitations

As the duration of the intervention is short for the immense changes in the organizational structure and processes, the positive effect of the intervention may be underestimated. Furthermore, the restricted budget allowed us to select only ten facilities in the intervention group. This implies that we could only involve some German states. As the final selection resides by the funder, selection bias could occur. In those ten facilities, the shadowing will be performed only in one of the living areas, so the transferability of the results to the whole facility may be restricted. The transferability of the customized concepts is limited as the number of participating nursing homes is restricted to ten. However, the prototypical concepts developed for the national rollout are open for individual adaptation and thus can be applied in every German nursing home.

## Data Availability

The resulting datasets will be available exclusively for scientific purposes from the principal and funding body GKV-Spitzenverband (Spitzenverband Bund der Krankenkassen—National Association of Statutory Health Insurance Funds) upon reasonable request.

## Abbreviations

DIP: Daily intervention planning (*tagesstrukturierte Interventionsplanung*)
EXQ: Employee Experience Questionnaire
GKV-SV: GKV-Spitzenverband (*Spitzenverband Bund der Krankenkassen*-National Association of Statutory Health Insurance Funds)
LTC: Long-term care
PeBeM: Acronym for the German word *Personalbemessung*, referring to the PeBeM1 study ‘Development of a scientifically based procedure for the standardized calculation of staffing requirements in long term-care’ and the PeBeM3, which is the subject of the study protocol presented here
QL: Qualification Level (*Qualifikationsniveau*)
QMM: Qualification mis model (*Qualifikations-Mix-Modell*)
